# Impact of cardiovascular risk factors and cardiac diseases on mortality in patients with moderate to severe ARDS: A retrospective cohort study

**DOI:** 10.1016/j.ijcrp.2024.200318

**Published:** 2024-08-10

**Authors:** Arnaud Gacouin, Pauline Guillot, Flora Delamaire, Alexia Le Corre, Quentin Quelven, Nicolas Terzi, Jean Marc Tadié, Adel Maamar

**Affiliations:** a CHU Rennes, Maladies Infectieuses et Réanimation Médicale, F-35033 Rennes, France; b Université Rennes 1, Faculté de Médecine, Biosit, F-35043 Rennes, France; c Inserm-CIC-1414, Faculté de Médecine, Université Rennes 1, IFR 140, F-35033 Rennes, France

**Keywords:** acute respiratory distress syndrome, Coronary artery disease, Atrial fibrillation, Valvular replacement, survival, Cohort study

## Abstract

**Background:**

Histor**y** of coronary artery disease (CAD) and/or atrial fibrillation (AF) and/or valvular replacement (VR) are prevalent among patients admitted to intensive care units (ICUs). The impact of these conditions on outcomes in patients with acute respiratory distress syndrome (ARDS) remains insufficiently explored.

**Methods:**

We performed a retrospective study on prospectively collected data from patients with ARDS and a PaO_2_/FiO_2_ ratio ≤150 mmHg. Patients were admitted between January 2006 and March 2022. We used multivariable logistic regression analysis. The primary outcome was 1-year mortality from admission to the ICU; secondary outcomes included mortality at 28 days and 90 days.

**Results:**

Among 1.033 patients, 181 (17.5 %) had a history of CAD and/or AF and/or VR. History of CAD and/or AF and/or VR was independently associated with 1-year mortality (Odds-Ratio (OR) = 2.59, 95 % confidence interval (CI) 1.76–3.82, p < 0.001), with mortality at 90 days (OR = 1.87, 95 % CI 1.27–2.76, p = 0.001), but not with mortality at 28 days (OR = 1.40, 95 % CI 0.93–2.11, p = 0.10). In sensitivity analyses, history of CAD and/or AF and/or VR remained independently associated with 1-year mortality in ICU survivors (OR = 3.58, 95 % CI = 2.41–7.82, p < 0.001).

**Conclusions:**

History of CAD and/or AF and/or VR was associated with mortality in ARDS. Prompt referral to cardiologists for comprehensive management post-ICU discharge may be warranted to optimize outcomes in this vulnerable population.

## Highlights

• History of coronary artery disease, atrial fibrillation, or valvular replacement is frequent in ARDS patients.
• These pre-existing cardiac diseases may worsen during ARDS.
• These cardiac diseases were retrospectively assessed for mortality in a large cohort of patients with ARDS.
• History of cardiac disease was independently associated with increased mortality at both 1 year and 90 days.

## 1 Introduction

In accordance with the Berlin definition for Acute Respiratory Distress Syndrome (ARDS), individuals with chronic heart disease may fulfill ARDS criteria if their respiratory failure is not entirely attributed to heart failure or fluid overload [1]. Notably, patients with chronic heart disease represent up to 15 % of patients admitted to the intensive care unit (ICU) for ARDS [2,3]. Furthermore, the presence of chronic heart disease has been linked to increased mortality among ARDS patients [2,3]. Severe infection, the most common etiology of ARDS, significantly influences the long-term outcome of patients with cardiovascular disease [4]. Chronic heart disease encompasses a broad spectrum of cardiac conditions, including coronary artery disease (CAD), heart rhythm disorders such as atrial fibrillation (AF), heart valvular disease, prior valvular replacement (VR). CAD, AF, and prior RV are identifiable cardiac conditions that may precede ICU admission. There is a notable scarcity of studies examining the impact of history of CAD, AF, and prior VR on outcomes of ARDS patients [5].

The aim of this study was study to investigate the association between prior history of CAD, AF, and VR with short- and long-term mortality in patients diagnosed with moderate to severe ARDS.

## 2 Material and Methods

### 2.1 Patients and study design

Since our ICUs participation in the multicenter clinical trial known as the ACURASYS study [6], we have been prospectively collecting data on patients with ARDS and PaO_2_/FiO_2_ ratio ≤150 mmHg. In addition to meeting hypoxia criteria, all patients exhibited acute respiratory failure and displayed new pulmonary parenchymal abnormalities on chest-X ray. We considered that hydrostatic edema was not the primary cause of respiratory failure based on results of pulmonary artery occlusion pressure measurements and/or echocardiographic assessment [1]. Follow-up information was obtained from medical records, the Civil Registration System of the city where the patients were born, and from the Institut national de la statistique et des études économiques (INSEE). Patients were enrolled from January 1, 2006 and March 1, 2022, which served as the end of the study period to ensure that all patients had at least 1 year of follow-up time. The study was conducted by the principles of the Helsinki Declaration and received approval from the hospital's ethic committee (Approval No. 21.105).

### 2.2 Data collection

We collected the following variables upon admission to the ICU: age, gender, Sequential Organ Failure Assessment (SOFA) [7], and simplified acute physiology score (SAPS) II [8]. SOFA was calculated on the first day of ARDS. The following major risk factors for cardiovascular disease were recorded: history of hypertension, obesity, diabetes mellitus, and active smoking. The other comorbidities collected were chronic obstructive pulmonary disease (COPD), liver cirrhosis, and immunosuppression. Patients were distinguished whether ARDS was due or not to infection, whether the origin of ARDS was thoracic or extra-thoracic, and whether PaO_2_/FiO_2_ ratio was or not ≤100 mmHg (i.e, severe ARDS). We also recorded the worst values of PaO_2_/FiO_2_ ratio, pH, and PaCO_2_ during the first day of ARDS. Initial ventilator settings and measures included respiratory rate, tidal volume per predicted body weight, plateau pressure, level of PEEP applied, respiratory system compliance, and ventilatory ratio [9]. Respiratory system compliance and ventilatory ratio were calculated using usual formula. The following treatments used in the ICU were recorded: steroids treatment within the first 7 days of admission, inotropic and vasopressor support, renal replacement therapy, prone positioning, extracorporeal membrane oxygenation (ECMO). At least, treatment limitation was noted.

### 2.3 Definitions

Patients were categorized based on whether they were older than 65 years or not. Obesity was defined as a body mass index (BMI) ≥ 30 kg.m-^2^. Immunocompromised patients were defined as those with aplasia, and/or recent chemotherapy for a solid tumor or hematologic disease. Severe hypercapnia was defined as a PaCO_2_ greater than or equal to 50 mmHg [10].

### 2.4 Exposure of interest

Our exposure of interest was as a previous history of CAD and/or AF, and/or VR. AF was considered present in patients admitted to the ICU with AF and in those who received long-term treatment for AF, even they were in normal sinus rhythm upon ICU admission. CAD was identified in patients who received treatments following confirmation by coronary angiography after cardiac catheterization or computed tomography. The primary outcome for the study was 1-year mortality from admission to the ICU while secondary outcomes included mortality at 90 days and 28 days from ICU admission.

### 2.5 Statistical analysis

Quantitative variables are described as median and interquartile range, while qualitative variable are presented as frequencies and percentages. Proportions were compared using the Chi-squared test, and continuous variables were compared using the Mann-Whitney *U* test. Survival curves were generated using the Kaplan-Meier method until 1 year from admission to the ICU and compared by the log-rank test. Multivariable logistic regression was employed to assess the association between co-morbid cardiac disease and mortality, adjusting for potential confounders. Results were expressed as Odds-Ratios (OR) with their 95 % confidence intervals (CI). The primary outcome, secondary outcomes, and sensitivity analyses were assessed with the same covariates as the primary outcome. Variables entered into the multivariable logistic regression were those significantly associated with 1-year mortality in the univariate analysis. To address multicollinearity issues, we examined the correlation using correlation matrix and selected the most comprehensive variables from those with high correlation. Pairwise interaction between AF and CAD was tested. After excluding variables highly correlated (Supplemental Fig. 1), the following variables were entered into multivariate models: SOFA score without respiratory points, age ≥65 years, obesity, liver cirrhosis, immunosuppression, PaO_2_/FiO_2_ ratio ≤100 mmHg, PaCO_2_ ≥ 50 mmHg, thoracic ARDS, infectious origin of ARDS, plateau pressure, renal replacement, and use of vasopressors. Additionally, the year of admission was entered in the multivariable to control the time effect. Additionally, we performed sensitivity analyses to assess the robustness of our results. To compare our findings with those of a previous study [5], we examined 1-year mortality in subgroups of patients with ARDS due to infection, in patients who survived their ICU stay, in patients with PaO_2_/FiO_2_ ratio ≤100 mmHg, and in patients of 50 years of age or older. For this last analysis, we did not adjust for age. All statistical analyses were performed using R 4.2.2 (R Foundation for Statistical Computing, Vienna, Austria). P values < 0.05 were considered statistically significant.

## 3 Results

### 3.1 Patients

During the 16-year period, a total of 1045 ARDS patients with a PaO_2_/FiO_2_ ratio ≤150 mmHg were admitted in our ICU, of which the 1-year status could be established for 1033 patients (see Flow chart, Supplemental Fig. 2). Among these patients, 181 (17.5 %) had prior history of CAD and/or AF and/or VR including 85 (8.2 %) with CAD, 89 (8.6 %) with AF, and 34 (3.3 %) with VR. Of the patients with CAD, 51 (4.9 %) had previously undergone coronary artery bypass surgery or stent placement. Among the 89 patients with prior history of AF, AF was present on admission to the ICU in 48 of them (4.8 %) and at least one episode of AF occurred during the ICU stay in an additional 16 patients (1.6 %). Thirteen patients (1.6 %) had both CAD and AF on admission to the ICU. Among the patient cohort, 394 patients (38.1 %) were 65 years or older, 378 patients (37 %) had diagnosed arterial hypertension, 236 patients (23 %) were active smokers, 357 patients (35 %) were obese, and diabetes mellitus was known in 145 patients (14 %). In the ICU, 360 patients (34.9 %) died. From admission to the ICU, 297 patients (28.7 %) were deceased by day 28, 388 patients (37.5 %) by day 90, and 439 (42.5 %) by 1 year. The frequency of treatment limitations decisions in the ICU did not significantly differ between patients with prior history of CAD and/or AF and/or VR and those without co-morbid cardiac disease (OR = 1.22, 95 % CI 0.59–2.36, p = 0.56) (Supplemental Fig. 3).

Patients with prior history of CAD and/or AF and/or VR were more likely to be older, male, and have hypertension and diabetes mellitus, as indicated in Table 1. The two groups did not significantly differ in terms of the respiratory parameters recorded on the first day of ARDS or the organ supports used in the ICU.

**Table 1:** Baseline characteristics, respiratory parameters the first day of ARDS, and life support stratified by history of coronary artery disease and/or atrial fibrillation and/or valvular replacement before admission to the ICU.

Variable	Prior coronary artery disease and/or atrial fibrillation and/or valvular replacement		P Value
	No (n = 852)	Yes (n = 181)	
Age, years	58.00 (47.00, 68.00)	67.00 (60.00, 75.00)	<0.001
Male gender, n (%)	532 (62.4)	146 (80.7)	<0.001
SAPSII score, points	46.00 (34.00, 63.00)	51.00 (38.00, 65.25)	0.012
SOFA score, points	9.00 (6.00, 12.00)	8.00 (6.00, 12.00)	0.68
Hypertension	285 (33.5)	93 (51.4)	<0.001
Obesity, n (%)	289 (33.9)	68 (37.6)	0.39
Diabetes mellitus, n (%)	103 (12.1)	42 (23.2)	<0.001
Active smoking, n (%)	210 (24.6)	26 (14.4)	0.004
COPD, n (%)	171 (20.1)	55 (30.4)	0.011
Liver cirrhosis, n (%)	72 (8.5)	10 (5.5)	0.24
Immunosupression, n (%)	140 (16.4)	20 (11.0)	0.09
Infectious origin^a^,^a^ n (%)	590 (69.2)	125 (69.1)	1.00
Pulmonary ARDS^b^,^b^ n (%)	740 (86.9)	161 (89.0)	0.52
PaO2/FiO2≤ 100 mmHg, n (%)	518 (60.8)	104 (57.5)	0.45
PaCO2≥ 50 mmHg, n (%)	485 (56.9)	106 (58.6)	0.53
pH	7.28 (7.18, 7.36)	7.28 (7.19, 7.35)	0.89
Respiratory rate, breaths/mn	27.00 (24.00, 30.00)	27.00 (24.00, 30.00)	0.46
Tidal volume/pbw, mL/kg	6.26 (6.00, 6.74)	6.31 (6.06, 6.61)	0.70
PEEP, cmH2O	10.00 (8.00, 12.00)	10.00 (8.00, 12.00)	0.68
Plateau pressure, cmH2O	26 (22,28)	25 (22,28)	0.51
Respiratory system compliance <20 ml/cmH2O	28.62 (22.70, 36.27)	30.33 (22.94, 37.25)	0.22
Ventilatory ratio	2.35 (1.87, 2.99)	2.41 (1.96, 3.00)	0.64
Prone positioning, n (%)	428 (50.2)	86 (47.5)	0.56
Renal replacement, n (%)	246 (28.9)	66 (36.5)	0.06
ECMO, n (%)	72 (8.5)	11 (6.1)	0.36
Treatment with steroids the first week of ARDS, n (%)	583 (68.4)	134 (74.0)	0.16
Vasopressors, n (%	736 (86.4)	162 (89.5)	0.31

Data are presented as median (interquartile range) for continuous measures.

ARDS, Acute Respiratory Distress Syndrome; ECMO, Extracorporeal Membrane Oxygenation; PEEP, Positive End-Expiratory Pressure; PBW, Predict Body Weight; SAPS, Simplified Acute Physiology Score; SOFA, Sequential Organ Failure Assessment; COPD, Chronic Obstructive Pulmonary Disease.

a Acute respiratory distress syndrome due to pneumonia or extra-thoracic infection.

b Acute respiratory distress syndrome due to direct thoracic injury.

### 3.2 One-year mortality

**Fig. 1:**
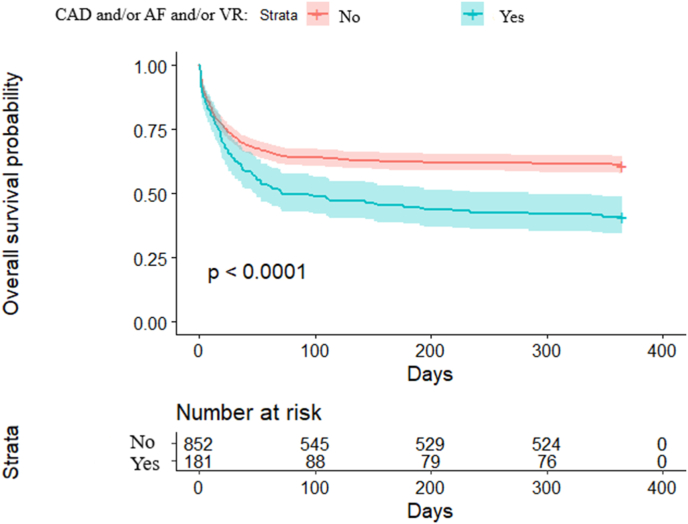
Kaplan-Meier survival stratified by previous history of coronary artery disease (CAD) and/or atrial fibrillation (AF), and/or valvular replacement (VR).

**Fig. 2:**
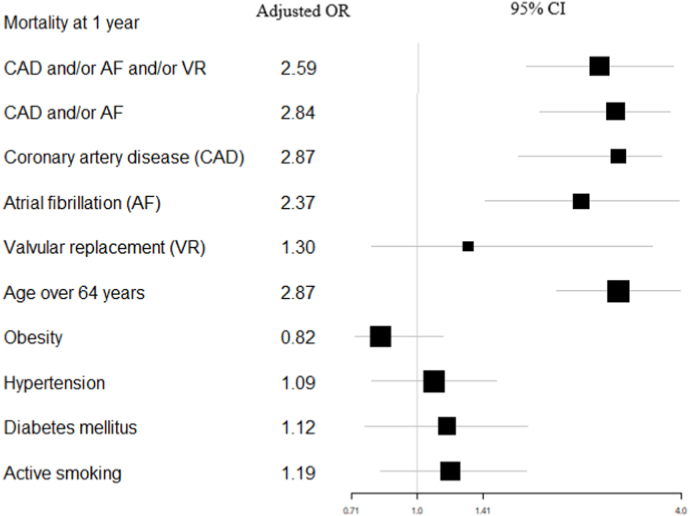
Forest plot for 1-year mortality from admission to the intensive care unit. Adjusted Odds-Ratios (ORs) and 95 % Confident Intervals (CIs) are shown on the figure.

At one year, mortality among patients with prior history of CAD and/or AF and/or VR was significantly higher (Fig. 1, p < 0.001 by log-rank). In unadjusted analyses, a prior history of CAD and/or AF and/or VR was associated with 1-year mortality (OR = 2.25, 95 % CI 1.63–3.13, p < 0.0001). Specifically, a prior history of CAD and/or AF (OR = 2.49, 95 % CI 1.78–3.54, p < 0.0001), a prior history of CAD (OR = 2.04, 95 % CI 1.31–3.23, p < 0.01), and a prior history of AF (OR = 2.35, 95 % CI 1.51–3.70, p < 0.001) were associated with increased mortality at 1 year. However, a prior history of VR (OR = 1.53, 95 % CI 0.77–3.08, p = 0.22) was not associated with mortality at 1 year. Extensive results for the unadjusted analyses for variables associated with 1-year mortality are l in the Supplemental Table 1. Notably, obesity was associated with a decreased risk of death (OR = 0.77, 95 % CI 0.59–0.99, p < 0.05). Conversely, hypertension was associated with increased risk of death (OR = 1.33, 95 % CI 1.03–1.72, p = 0.03). After multivariable adjustment, the risk of 1-year mortality remained significantly higher in patients with history of CAD and/or AF and/or VR (OR = 2.59, 95 % CI 1.76–3.82, p < 0.001). Specifically, history of CAD and/or AF was independently associated with 1-year mortality (OR = 2.84, 95 % CI 1.90–4.23, p < 0.001). Additionally, prior history of CAD and prior history of AF were both individually associated with mortality at 1 year (Fig. 2). With respect to risk factors for cardiovascular disease, only age ≥65 years remained independently associated with 1-year mortality (OR = 2.87, 95 % CI 2.08–3.99, p < 0.01).

**Fig. 3:**
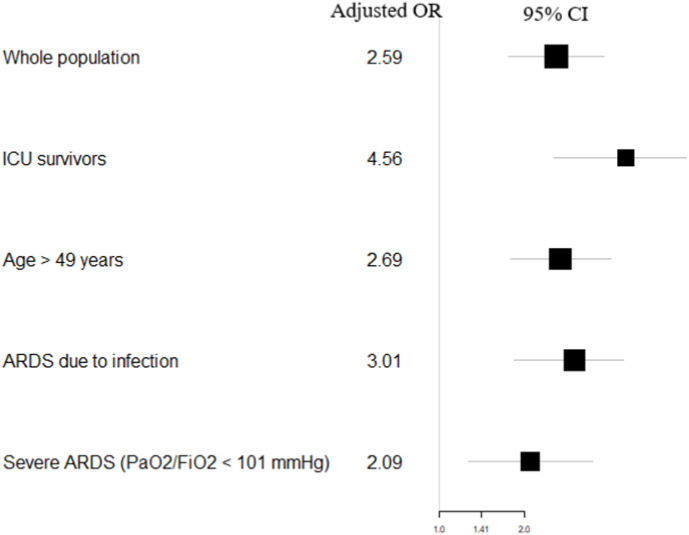
Forest plot for 1-year mortality of variables used for sensitivity analyses. Adjusted Odds- Ratios (ORs) and 95 % confident intervals (CIs) were calculated for prior history of coronary artery disease and/or atrial fibrillation, and or valvular replacement.

In a sensitivity analysis restricted to patients who survived their ICU stay, a prior history of CAD and/or AF and/or VR was independently associated with 1-year mortality (OR = 4.56, 95 % CI 2.54–8.14, p < 0.001) (Fig. 3). Additionally, prior history of CAD and/or AF and/or VR remained independently associated with 1-year mortality in patients aged 50 years or older (OR = 2.59, 95 % CI 1.78–4.07, p < 0.001), in patients with ARDS due to pulmonary or extra-pulmonary infection (OR = 3.01, 95 % CI 1.84–4.91, p < 0.01), and in patients with severe ARDS (OR = 2.09, 95 % CI 1.27–3.49, p < 0.001). Furthermore, there was no significant interaction between CAD and AF (p = 0.33).

### 3.3 Secondary outcomes

In unadjusted analyses, a prior history of CAD and/or AF and/or VR (OR = 1.80, 95 % CI 1.28–2.43, p < 0.001), CAD and/or AF (OR = 1.90, 95 % CI = 1.36–2.67, p < 0.001), CAD (OR = 1.64, 95 % CI 1.05–2.50, p = 0.03), and AF (OR = 1.72, 95 % CI 1.11–2.68, p = 0.01), were associated with mortality at day 90. However, a prior history of VR was not associated with mortality at day 90 (OR = 0.92, 95 % CI 0.36–2.16, p = 0.67). Furthermore, a prior history of CAD and/or AF and/or VR, a prior history of CAD and/or AF, CAD, or AF alone were not associated with mortality at day 28 (Supplemental Fig. 4).

After adjustment, a prior history of CAD and/or AF and/or VR, a prior history of CAD and/or AF, and a prior history of CAD remained independently associated with mortality at day 90 (OR = 1.87, 95 % CI 1.27–2.76, p = 0.001), but not at day 28 (OR = 1.40, 95 % CI 0.93–2.11, p = 0.10). None of the variables was independently associated mortality at day 28. None of the risk factors for cardiovascular disease was associated with mortality at day 28 and day 90 (Supplemental Fig. 5). Overall, adjusted OR for death increased with time (Supplemental Fig. 6).

## 4 Discussion

In this retrospective cohort study of patients with moderate to severe ARDS, we investigated the impact of major risk factors for cardiovascular diseases and prior history of CAD, AF, and VR on short- and long-term survival. We found that 17.5 percent of patients had a history or CAD and/or AF and/or VR, and these patients were more likely to be older and to have hypertension and/or diabetes mellitus. History of CAD and/or AF and/or VR was independently associated with mortality at 1 year, and the independent association persisted in sensitivity analyses, particularly within the subgroup of ICU survivors. Furthermore, we found that CAD and AF were independently associated with 1-year mortality, whereas history of VR did not show a significant association. Additionally, the impact of prior history of CAD and/or AF and/or VR on survival was also noted at 90 days from admission to the ICU, although this association was not significant at 28 days. Among the risk factors for cardiac disease assessed in this study, only age ≥65 years was independently associated with mortality at 1 year. To our knowledge, this is the largest study to assess the impact of the most frequent cardiac diseases and major risk factors for cardiac disease on both short-and long-term survival in ARDS patients.

Previously, authors demonstrated that cardiovascular risk factors and cardiac disease were associated with mortality in critically ill patients, regardless of whether or not they had ARDS [11]. For instance, ventricular arrhythmia and cardiogenic shock are associated with an increased risk of death [12,13]. Acute respiratory failure is recognized as a risk factor for new-onset AF, and several studies have reported that new-onset AF during ICU stay is associated with worse outcomes [13,14]. For instance, Ambrud DB et al. [14] found that new-onset AF during ARDS was associated with increased 90-day mortality. Despite the high prevalence of AF in critically ill patients, to our knowledge, the impact of a history of AF on the outcome of ARDS patients has not been previously assessed. Conversely, the impact of CAD on outcomes of ARDS patients has been recently assessed [5]. In a pooled population of ARDS patients from four randomized trials, it was found that ischemic heart disease was associated with 60-day mortality, with an OR of 1.75 (95 % CI 1.06 to 2.85). Remarkably, there is limited existing literature on this topic, highlighting the novelty of our study. Our findings corroborate the results previously published by Biondi M et al. [5]. It is noteworthy that a prior history of VR was not associated with survival, although this finding should be interpreted cautiously due to the small number of patients analyzed.

A significant proportion of individuals who have undergone ICU treatment subsequently develop cognitive, psychiatric and/or physical disabilities. This syndrome, which has been given the designation of post-intensive care syndrome, is recognized as a consequence of the treatment received in the ICU. In patients who have survived ARDS, cognitive impairment is observed in 46 %–80 % of cases at one year. The incidence of ICU-acquired weakness is estimated to be around 45 %–50 % [15].The effects of ARDS are not short-lived, and there is growing body of evidence suggesting that a period of critical illness due to ARDS is frequently associated with the onset of chronic health problems, especially in cases of sepsis-induced ARDS [[15], [16], [17]]. Several processes may contribute to the exacerbation of pre-existing structural heart resulting from a history of CAD, AF, or valvular disease.

Firstly, ARDS patients receive lung-protective ventilation, which can result in CO_2_ retention and respiratory acidosis. Hypercapnic acidosis is associated with coronary vasodilatation [18], however, it can also lead to reduced cardiac contractile function and increased ventricular stroke work, as observed in animal model [19]. Secondly, ARDS patients experience hypoxemia, and oxygenation goals are set low during lung-protective ventilation. Consequently, these patients are at increased risk of myocardial hypoxia. While chronic hypoxia is known to contribute to the development of atherosclerosis, hypertension, and heart failure [19], the impact of acute hypoxia on pre-existing myocardial lesions is less established. Several experimental studies suggest that acute hypoxia may trigger the secretion of various factors involved in myocardial remodeling [20]. Additionally, it is well-established that ARDS is associated with acute lung and systemic inflammation [21]. The relationship between inflammation and heart function is complex. Chronic inflammation is known to play a causative role in the development of cardiovascular diseases, particularly atherosclerosis. However, the long-term consequences of acute inflammation on myocardial health are not well defined. Nonetheless, some data suggest that acute inflammation may accelerate myocardial damage in patients with CAD [22,23]. Lastly, heart-lung interactions undergo significant alterations during mechanical ventilation in ARDS patients [24], characterized by an increase in pulmonary arterial pressure and pulmonary vascular resistance,. Consequently, a large proportion of patients experience right ventricular dysfunction.

Our findings suggest that the detrimental effect of a prior history of CAD and AF on ARDS outcomes become evident only after 28 days and escalate over time. The long-term consequences of ARDS on myocardial function remain largely unknown. However, sepsis is well-established as a precipitating factor of cardiovascular disease [4], and sepsis is prevalent among ARDS patients, particularly due to pneumonia being a leading cause of ARDS. A large retrospective cohort study demonstrated that survivors of sepsis face an increased risk of major adverse cardiovascular events within 1 year after hospital discharge [25].

An important finding of the study is that previous history of CAD and/or AF and/or VR remained independently associated with 1-year mortality in ICU survivors. A recent systematic review by authors [26] revealed that up to 80 % of critical care survivors may encounter challenges with medication management during their recovery. Patients with risk factors for cardiovascular disease, as well as those with established cardiovascular diseases such as CAD and AF, often, often require long-term treatments. However, some of these treatments, particularly antihypertensive medication, are frequently interrupted during the ICU stay. This practice may have a significant impact on cardiac remodeling. On other hand, some treatments are inappropriately continued while the heart condition has changed with ARDS. None of the other studied risk factors for cardiovascular disease that are amenable to treatment were independently associated with 28-, 90-, and 1-year mortality. However, 1-year mortality was higher in patients with hypertension in the unadjusted analysis, suggesting that ARDS patients with hypertension require a particular attention after ICU discharge. Chronic hypertension can have significant consequences on various organs, particularly the heart and kidneys. It is reasonable to hypothesize that patients with history of arterial hypertension are at heightened risk of deteriorating cardiac or renal function during and after the period of ARDS, particularly among those who develop shock. Obesity was associated with a better 1-year survival in the unadjusted analysis. Patients with obesity are at an increased risk for developing ARDS [27], and obesity is a risk factor for diabetes, hypertension, and heart disease [28]. Active smoking, a risk factor for cardiovascular disease and ARDS [29] was less frequent in patients with prior history of CAD, AF, or VR compared to patients without these diseases.

## 5 Limitations

In addition to its retrospective nature, our study has several limitations. We did not collect prospectively results of echocardiography performed around admission to the ICU. Consequently, the left ventricular ejection fraction of patients at admission to the ICU was not available. Except previous VR, we did not assess the impact of valvular disease. We did not collect the occurrence of cardiovascular events during the ICU stay, including onset of AF and modifications of electrocardiogram suggestive of myocardial infarction. We determined the cause of death in the ICU but were unable to determine the cause of death for the patients who died during the follow-up period and after ICU discharge. While patients were included over a long period, we acknowledge the possibility that changes in medical practices over time could have influenced the results. Indeed, the results of randomized studies indicated an increase in the use of early and prolonged prone positioning [30] and extracorporeal membrane oxygenation [31] in our ICU during the period of the study. However, we adjusted for the time period of the study in our analyses. Additionally, we did not assess changes in cardiovascular treatments associated with the ICU stay.

## Conclusions

In our study of patients with moderate to severe ARDS, we found that history of CAD and/or AF and/or VR was independently associated with increased mortality at 1-year, a trend that persisted in ICU survivors. Similar associations were observed for survival at 90 days, though not at 28 days. These results underscore the importance of recognizing and addressing cardiovascular comorbidities in ARDS patients, particularly those with CAD and/or AF. Prompt referral to cardiologists for comprehensive management post-ICU discharge may be warranted to optimize outcomes in this vulnerable population.

## Declaration of interests

All of the authors have no conflicts of interest.

## Funding sources

This research did not receive any specific grant from funding agencies in the public, commercial, or not-for- profit sectors.

## CRediT authorship contribution statement

**Arnaud Gacouin:** Writing – review & editing, Methodology, Formal analysis, Data curation, Conceptualization. **Pauline Guillot:** Writing – original draft, Data curation, Conceptualization. **Flora Delamaire:** Writing – original draft, Supervision, Data curation, Conceptualization. **Alexia Le Corre:** Methodology, Data curation, Conceptualization. **Quentin Quelven:** Data curation. **Nicolas Terzi:** Writing – review & editing, Validation, Supervision. **Jean Marc Tadié:** Writing – review & editing, Supervision, Conceptualization. **Adel Maamar:** Writing – original draft, Supervision, Methodology, Conceptualization.
