# Supplementary material for: Impact of cardiovascular risk factors and cardiac diseases on mortality in patients with moderate to severe ARDS: A retrospective cohort study
Source: Int J Cardiol Cardiovasc Risk Prev. 2024 Aug 10;22:200318. doi: 10.1016/j.ijcrp.2024.200318 (PMC11372786; doi:10.1016/j.ijcrp.2024.200318)
Supplement: Multimedia component 1 [file mmc1.docx]

Supplemental material

**Impact of coronary artery disease and or atrial fibrillation on the outcomes of patients with moderate to severe acute respiratory distress syndrome**

Arnaud Gacouin, Pauline Guillot, Flora Delamaire, Alexia Le Corre, Quentin Quelven, Nicolas Terzi, Jean Marc Tadié, and Adel Maamar.

.

**Supplemental Figure 1**

**CORRELATION MATRIX**


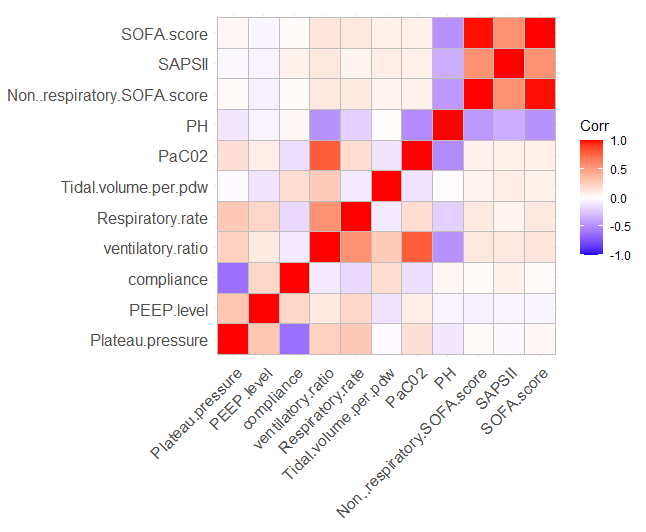


Supplemental Figure 1 : Correlation matrix for continuous variables.

**Supplemental Figure 2**

**FLOW CHART**

**
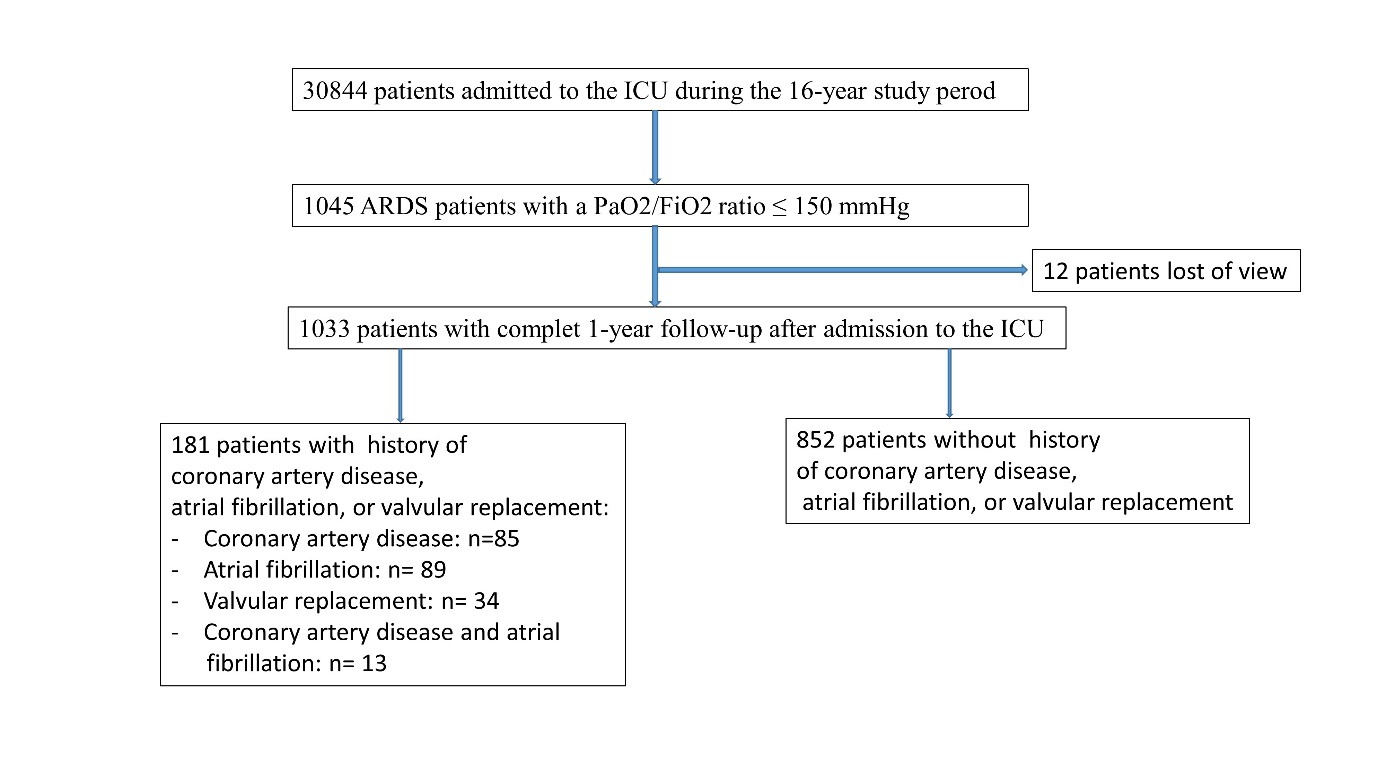
**

Supplemental Figure 2 : Flow chart.

**Supplemental Figure 3**

**TREATMENT LIMITATIONS**

**
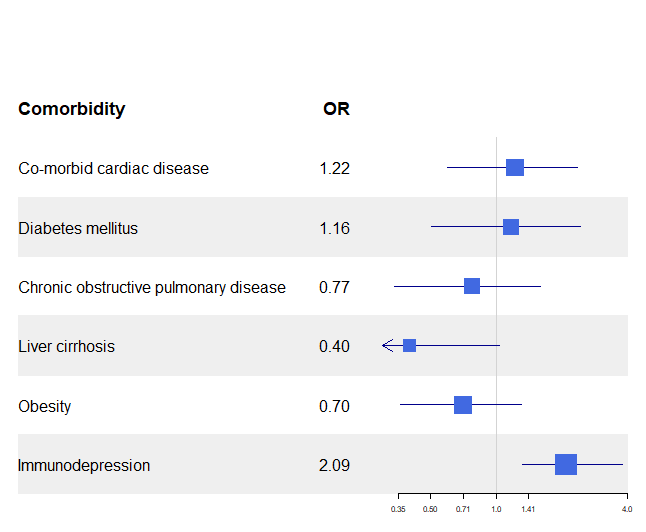
**

Supplemental Figure 3: Forest plot for treatment limitations in the ICU. Co-morbid cardiac disease means prior history of coronary artery disease and/or atrial fibrillation and/or history of valvular replacement.

**Supplemental Figure 4**

**MORTALITY AT DAY 28 AND DAY 90: UNADJUSTED ANALYSIS**

**
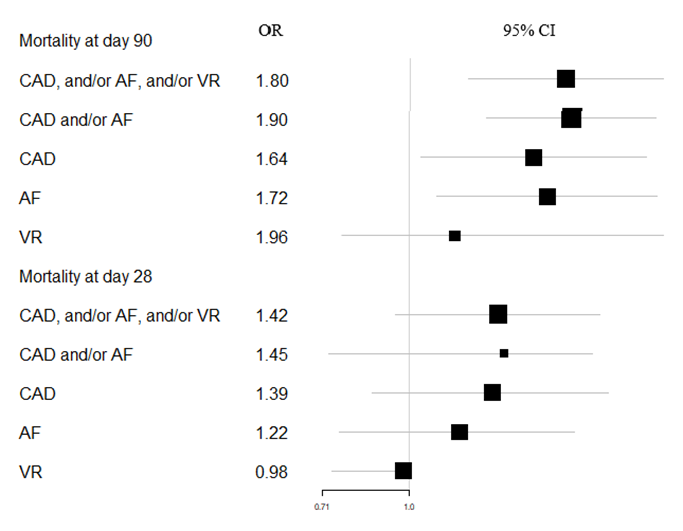
**

Supplemental Figure 5: Forest plot unadjusted analyses for mortality at day 28 and at day 90.; OR, Odds-Ratio; CI, Confident Interval; CAD: coronary artery disease; AF, atrial fibrillation; VR, valvular replacement.

**Supplemental Figure 5**

**MORTALITY AT DAY 28 AND DAY 90: ADJUSTED ANALYSIS**

**
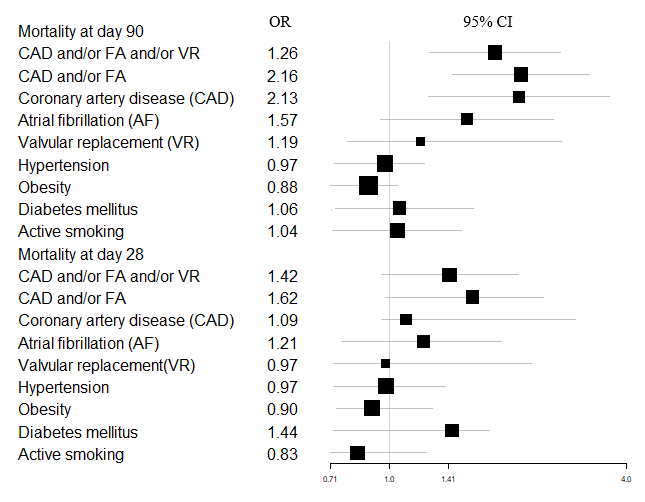
**

Supplemental Figure 6: Forest plot for mortality at 28 days and 90 days from admission to the intensive care unit. Adjusted Odds-Ratios (ORs) and 95% Confident Intervals (CIs) are shown on the figure.

**Supplemental Figure 6**

**MORTALITY: ODDS RATIOS WITH TIME**

**
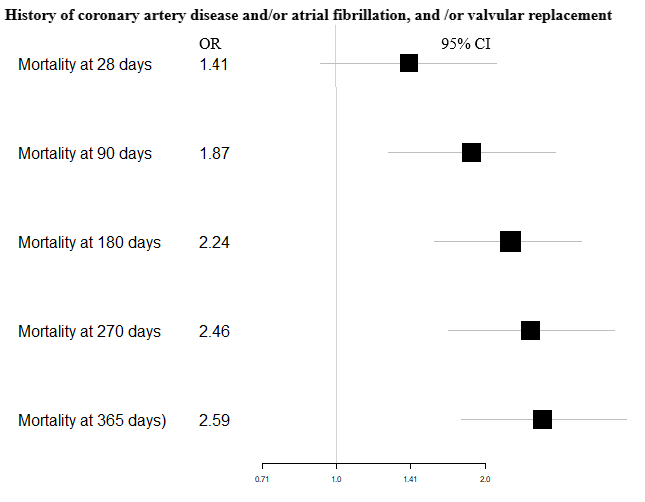
**

Supplemental figure 6: Forest plot adjusted analyses for mortality at 28, 90, 180, 270, and 365 days from admission to the ICU. OR, Odds-Ratio; CI, Confident Interval.

**Supplemental Table 1**

Supplemental table 1: unadjusted analysis for 1-year mortality

| Variable | Odds Ratio | 95% Confident Interval | P Value |
| --- | --- | --- | --- |
| Prior history of CAD and/or AF and/or VR | 2.25 | 1.63-3.13 | <0.0001 |
| Prior history of CAD and/or AF | 2.49 | 1.78-3.74 | <0.001 |
| Prior history of CAD | 2.04 | 1.31-3.23 | <0.01 |
| Prior history of AF | 2.35 | 1.51-3.70 | <0.001 |
| Prior history of VR | 1.53 | 0.77-3.08 | 0.22 |
| Hypertension | 1.33 | 1.03-1.72 | 0.03 |
| Age ≥ 65 years | 1.99 | 1.55-2.58 | <0.001 |
| Male gender | 1.24 | 0.96-1.61 | 0.11 |
| SAPS II score (1 point increment) | 1.04 | 1.03-1.05 | <0.001 |
| SOFA score (1 point increment) | 1.23 | 1.19-1.28 | <0.001 |
| SOFA score without respiratory points (1 point increment) | 1.24 | 1.19-1.28 | <0.001 |
| Obesity | 0.77 | 0.59-0.99 | 0.046 |
| Diabetes mellitus | 1.19 | 0.93-1.69 | 0.34 |
| Active smoking | 0.90 | 0.67-1.21 | 0.50 |
| COPD | 0.97 | 0.72-1.31 | 0.85 |
| Liver cirrhosis | 2.83 | 1.77-4.59 | <0.001 |
| Immunosuppression | 3.38 | 2.38-4.87 | <0.001 |
| ARDS due to infection | 0.54 | 0.41-.70 | <0.001 |
| Pulmonary ARDS | 0.29 | 0.19-0.43 | <0.001 |
| PaO_2_/FiO_2_ ≤ 100 mmHg | 1.55 | 1.20-1.99 | <0.001 |
| PaCO_2_ ≥ 50 mmHg | 1.41 | 1.10-1.82 | <0.01 |
| pH < 7.35 | 2.12 | 1.57-2.88 | <0.001 |
| Respiratory rate (1 cycle increment) | 1.03 | 1.001-1.06 | 0.048 |
| Tidal volume per pbw (1 ml/kg increment) | 1.17 | 1.03-1.33 | 0.02 |
| PEEP (1 cmH_2_O increment) | 0.92 | 0.89-0.96 | <0.001 |
| Plateau pressure (1 cmH_2_O increment) | 1.17 | 1.03-1.33 | 0.02 |
| Respiratory system compliance, (1 ml/cmH_2_O decrement) | 0.98 | 0.97-0.99 | 0.03 |
| Ventilatory ratio | 1.26 | 1.09-1.46 | <0.01 |
| Prone positioning | 0.78 | 0.62-1.01 | 0.06 |
| Renal replacement | 3.96 | 2.99-5.26 | <0.001 |
| ECMO | 1.49 | 0.95-2.35 | 0.08 |
| Treatment with steroids the first week of ARDS | 1.27 | 0.98-1.66 | 0.09 |
| Treatment with vasopressors | 3.08 | 2.03-4.82 | <0.001 |

Definition of abbreviations: AF, Atrial Fibrillation; ARDS, Acute Respiratory Distress Syndrome; ECMO, Extracorporeal Membrane Oxygenation; CAD, Coronary Artery Disease; COPD, Chronic Obstructive Pulmonary Disease; PBW, Predicted Body Weight; PEEP, Positive-End Expiratory Pressure; SAPS, Simplified Acute Physiology Score; SOFA, Sequential Organ Failure Assessment; VR, valvular replacement.
